# Low Levels of Influenza Vaccine Uptake among the Diabetic Population in Spain: A Time Trend Study from 2011 to 2020

**DOI:** 10.3390/jcm11010068

**Published:** 2021-12-23

**Authors:** Jose J. Zamorano-Leon, Rodrigo Jimenez-Garcia, Ana Lopez-de-Andres, Javier de-Miguel-Diez, David Carabantes-Alarcon, Romana Albaladejo-Vicente, Rosa Villanueva-Orbaiz, Khaoula Zekri-Nechar, Sara Sanz-Rojo

**Affiliations:** 1 Department of Public Health & Maternal and Child Health, Faculty of Medicine, Universidad Complutense de Madrid, IdISSC, 28040 Madrid, Spain; jjzamorano@ucm.es (J.J.Z.-L.); anailo04@ucm.es (A.L.-d.-A.); dcaraban@ucm.es (D.C.-A.); ralbadal@ucm.es (R.A.-V.); mrvillan@med.ucm.es (R.V.-O.); 2 Respiratory Department, Hospital General Universitario Gregorio Marañón, Instituto de Investigación Sanitaria Gregorio Marañón (IiSGM), Universidad Complutense de Madrid, 28040 Madrid, Spain; javier.miguel@salud.madrid.org; 3 Department of Medicine, Faculty of Medicine, Universidad Complutense de Madrid, IdISSC, 28040 Madrid, Spain; kzekri@ucm.es; 4 Faculty of Health Science, Universidad Alfonso X El Sabio, Villanueva de la Cañada, 28691 Madrid, Spain; sara.sanz.rojo@gmail.com

**Keywords:** influenza seasonal, vaccine coverage, diabetes, uptake, trend, Spain

## Abstract

(1) Background: In this work, we aim to describe influenza vaccine uptake among the diabetic population in Spain to assess the time trend from 2011 to 2020 and identify predictors of vaccine uptake among diabetes patients. (2) Methods: We conducted a descriptive cross-sectional study using the European Health Interview Survey for Spain (2014 and 2020) and the Spanish National Health Surveys (2011 and 2017). The independent variables analysed included socio-demographic characteristics, health-related variables and lifestyle variables. We matched each participant with diabetes with a non-diabetic participant based on age, sex, place of residence and year of survey. (3) Results: The overall coverage among diabetic adults was 52.1% compared to 40.6% for matched participants without diabetes (*p* < 0.01). The vaccine uptake among adults with diabetes was 52.6% in 2011, 54.38% in 2014 and 53.4% in 2017. The adjusted OR of having been vaccinated in 2020, with respect to 2011, was not significant at 0.87 (95% CI: 0.72–1.06). Factors such as being male, higher age, being affected by respiratory disease or cancer and being physically active were identified as positive predictors for influenza vaccination uptake, while smoking was a negative predictor. (4) Conclusions: The influenza vaccine uptake is below desirable levels among the adult diabetic population in Spain and has not improved from 2011 to 2020. More efforts should be made to increase influenza vaccine uptake in this high-risk group, especially for women, those aged 18–64 years, without other high-risk conditions and smokers.

## 1. Introduction

Influenza is a respiratory disease with a high impact on public health and the economy in terms of health system costs and its effect on the workforce [1]. A number of studies have demonstrated how influenza vaccination decreases absenteeism in the workplace, hospitalizations and mortality [2,3]. The World Health Organization (WHO) has implemented the Global Influenza Program for the development of vaccines and flu pandemic guidelines [4]. However, despite constant efforts by governments and institutions, influenza vaccine uptake is far below the recommended 75% among older people and patients with chronic illnesses, as proposed by WHO [5]. In Europe, for the 2013–2014 and 2014–2015 influenza seasons, the average vaccination rate was 45.5% and 49.8% among the elderly and patients with chronic medical conditions, respectively [6]. In Spain, the vaccination rate among high-risk groups (aged ≥ 65 years, patients with chronic medical conditions, pregnant women and socio-sanitary workers) was 54.7% during the 2019–2020 vaccination campaign [7].

Influenza vaccination is strongly recommended for patients diagnosed with diabetes mellitus. Influenza infection in the diabetic population is related to higher risks of hospital and intensive care unit admissions and higher chances of needing respiratory support and mortality [8,9,10]. The body of evidence about influenza vaccination in diabetic patients points out the protective effects of the vaccine against pneumonia [11], hospitalizations due to influenza virus infection and all-cause mortality [12,13,14]. Despite the above-mentioned benefits of influenza vaccination among the diabetic population, influenza vaccination uptake in persons with diabetes is lower than desirable [15,16,17]. In 2017 in Spain, influenza vaccination for those aged 65 years or over was around 62%, while it only reached 51.5% in the 15–64 age group [18,19]. Unfortunately, in a recent Spanish study among persons with diabetes, no significant improvement in influenza vaccine uptake was found from 2014 to 2017 [19]. Fear of potential side effects and previous negative experiences with vaccination are considered as classical reasons for refusing vaccination [20]. However, socio-demographic and health-related variables also seem to be closely associated with adherence to influenza vaccination in diabetic populations [19,20,21,22,23].

There are also novel factors that may affect adherence to influenza vaccination. The COVID-19 pandemic has raised concerns about vaccination campaigns for influenza due to lockdowns, high sanitary services demands and limitations on human interactions. In the case of healthcare workers, the pandemic served as a vaccination incentive, likely because this vaccine has been associated with lower chances of SARS-CoV-2 infections and hospital admissions [24,25,26]. All the above-mentioned makes it even more important to analyse influenza vaccine uptake rates among high-risk populations, such as diabetes patients, as well as to identify factors associated with adherence to influenza vaccination.

Using data from the 2011 and 2017 Spanish National Health Surveys (SNHS) and the 2014 and 2020 European Health Interview Survey for Spain (EHS), we aim to describe the coverage of influenza vaccination in the adult diabetic population living in Spain, to assess the time trend from 2011 to 2020 and to identify predictors of vaccine uptake among diabetic patients.

## 2. Materials and Methods

### 2.1. Study Design and Study Population

We conducted a descriptive cross-sectional study based on data obtained from the EHS 2020. The EHS 2020 is a home-based, personal interview examining a nationwide, representative sample of the civilian, non-institutionalized population aged 15 years or over, residing in main family dwellings (households) in Spain. This survey uses a three-stage stratified sampling, and 22,188 subjects were selected from 15 July 2019 to 24 July 2020. However, after 17 March 2020, interviews became telephonic due to the COVID-19 pandemic. More information about the EHS 2020 methodology is described online [27].

To analyse the time trend in influenza vaccination uptake from 2011 to 2020, we consulted the databases of the Spanish National Health Surveys (SNHS) conducted in 2011 (SNHS 2011–2012) and 2017 (SNHS 2017) as well as the EHS 2014. The methods of the SNHS are identical to those used in EHS. For the purpose of our study, only survey respondents aged 18 years or over were included.

More details on the methodologies of these four surveys can be found elsewhere [28,29,30].

### 2.2. Matching Method

After collecting all information from subjects who participated in the surveys in the years 2011–2012, 2014, 2017 and 2020, we matched the diabetic and non-diabetic populations. This matching was performed to reduce bias due to differences in the distribution by age, gender and place of residence between the diabetic and non-diabetic populations.

We performed one-by-one matching. This means, for example, that for a woman with diabetes interviewed in the SNHS 2011, aged 50 years and living in the autonomous community of Madrid, we selected a woman not reporting diabetes interviewed in the SNHS 2011, aged 50 years and living in the autonomous community of Madrid. We carried out this process for every single survey respondent with diabetes. If more than one non-diabetic subject was available for a subject with diabetes, the selection was performed randomly. After matching, a total of 7369 pairs were included in the final analysis, and the distribution according to age and gender was identical for those with and without diabetes.

### 2.3. Study Variables

The variables used were identically collected in all the surveys used.

The dependent variable “Influenza vaccine uptake” was considered positive when subjects answered affirmatively to the question “Were you vaccinated against influenza during the last vaccination campaign?”

The independent variables were classified into three groups: (1) socio-demographic variables (“gender”, “age groups”, “social status”, “marital status”, “education level”), (2) health-related variables (“self-rated health status”, “Body Mass Index”, “myocardial infarction”, “respiratory diseases”, “cancer”, “stroke”, “diabetes mellitus”) and finally, (3) lifestyle-related variables (“alcohol intake”, “smoking habits”, “physical activity”). The aforementioned pathologies are medical conditions for which influenza vaccination is recommended in Spain. These conditions were considered “present” when participants answered affirmatively to the question “Have you been diagnosed by a practitioner with this disease?” Detailed descriptions and categories for these variables can be found in Table S1.

### 2.4. Statistical Analysis

We performed a descriptive analysis providing absolute frequencies, percentages and 95% confidence intervals (95% CI). The proportion of vaccine uptake was estimated according to study variables for the diabetes and non-diabetes groups. These vaccine uptakes were compared using the Chi-squared test.

Multivariable logistic regression models were constructed to identify independent predictors of adherence to influenza vaccination among the diabetic population included in the EHS 2020.

To assess the time trend in vaccination rates from 2011 to 2020, we joined all paired databases and developed a logistic regression model adjusted by gender, age groups and year of the survey. In this model, the dependent variable was influenza vaccine uptake.

In the multivariable models, we included variables with a significant association in the univariate analysis or those reported as relevant in the literature. Odds ratios (OR) with 95% CI were provided. The Hosmer–Lemeshow test was used to assess the goodness of fit for the regression models constructed. A statistical analysis was performed using the software SPSS v25.0 (IBM Corp. Released 2017. IBM SPSS Statistics for Windows. Armonk, NY, USA). A two-tailed *p*-value < 0.05 was considered statistically significant.

### 2.5. Ethical Aspects

According to the Spanish legislation, an ethics committee approval is not required when public, free access datasets with anonymous data such, as the EHS and the SNHS, are used for investigation. These datasets can be downloaded from the Spanish Ministry of Health′s webpage [31,32].

## 3. Results

### 3.1. Characteristics of the Participants with Diabetes in the EHS 2020

From the 15,622 subjects aged ≥18 years included in the EHS 2020 database; a total of 2049 people were identified as diabetes patients. Gender distribution was 49.6% female and 50.4% male. As expected, the majority of the diabetic patients were older than 64 years (68.5%). Table S2 shows the distribution according to socio-demographic characteristics and health-related variables for 2049 matched pairs of diabetic and non-diabetic subjects. The diabetic population had lower social status, worse self-rated health status and higher education levels when compared to the matched population without diabetes. Diabetes patients had higher proportion of obesity and absence of physical activity as well as less alcohol consumption than non-diabetic participants (Table S2). Finally, more diabetes patients reported comorbid chronic conditions such as myocardial infarction (7.3% vs. 4.1%), respiratory diseases (12.8% vs. 9.5%) and stroke (6.2% vs. 3.3%) than matched non-diabetes subjects (Table S2).

### 3.2. Trends from 2011 to 2020 in the Influenza Vaccination Uptake among the Spanish Diabetic Population

Shown in Table 1 are the vaccine uptakes according to gender and age groups for diabetic and matched non-diabetic participants in each of the four surveys analysed. The results revealed that the total uptake was higher in male gender, and among those aged 75 years or over for the diabetic (53.8%; 68.6%) and the non-diabetic population (43.9%; 64.2%). The influenza vaccination uptake was higher among those with than without diabetes in the four surveys analysed, and when we stratified the analysis by gender and age groups, the proportion of adults with diabetes who reported having been vaccinated was similar in the years 2011 (52.6%), 2014 (54.3%), 2017 (53.4%) and 2020 (53.1%). The adjusted OR of having been vaccinated in 2020 with respect to 2011 was not significant (0.87 95% CI: 0.72–1.06), showing no change over time in the uptake for the total diabetic population.

### 3.3. Analysis of Influenza Vaccine Uptake for Participants with Diabetes in the EHS 2020

The vaccine uptake for participants with diabetes in the EHS 2020 and their matched non-diabetic pairs are shown in Table 2.

For all the categories of the study variables, diabetic participants showed significantly higher vaccine uptake compared to non-diabetic subjects, except for those who had reported myocardial infarction or respiratory diseases.

According to our univariate analyses in Table 1 and Table 2, the categories for each variable with the highest values of influenza vaccination uptake among the diabetic population of the EHS 2020 were aged 75 years or over, male gender, marital status of “separated, divorced, or widowed”, primary education level, medium social status, obesity, no alcohol consumption, no smoking, no physical activity, “very good or good” self-rated health, and reporting having suffered myocardial infarction, respiratory diseases, cancer or stroke.

### 3.4. Multivariable Analysis of Influenza Vaccine Uptake for the Spanish Diabetic Population

Table 3 reveals the predictors of influenza vaccination among adults with diabetes collected in the 2011, 2014, 2017 and 2020 surveys after logistic regression analysis. Diabetic subjects aged 75 years or over were 6.753 times more likely to report being vaccinated than those aged 18–49 years. Female gender was associated with lower vaccine uptake, OR 0.872 (95% CI 0.772–0.982)

Table 4 shows the results of the multivariable logistic regression analysis to identify the predictors of influenza vaccination among the participants with diabetes in the EHS 2020. Results revealed that positive predictors for influenza vaccination were age 65 years or over, having suffered respiratory diseases or cancer and being physically active. Smoking was identified as a negative predictor for influenza vaccination.

## 4. Discussion

In the present study, we analysed the influenza vaccination rate of the diabetic population based on the EHS 2020. In addition, we analysed the time trend from 2011 to 2020 in Spain, revealing that uptake among adults with diabetes was 52.1% in 2020, and this uptake did not improve from 2011.

Our study showed that in the EHS 2020S, the uptake was 1.3-fold higher among adult diabetic participants when compared to sex- and age-matched non-diabetic individuals.

Epidemiological studies conducted in European countries have also reported higher vaccine uptake among those with than without diabetes [33,34]. The vaccine uptake of 52.1% found in people with diabetes in the EHS 2020 is similar to previous studies conducted on diabetic populations in Spain, which reported vaccination rates ranging from 43% to 57% [21,22]. However, it is still far from the goal for seasonal influenza vaccination rate of 90% for high-risk adults (including diabetic patients) as recommended by the U.S. Healthy People 2020 Objectives [35].

Compared with other European countries, Spain remains in an intermediate position. In Portugal, Machado et al. reported a vaccination uptake of 43.8% in diabetic subjects [36], while for Denmark, vaccine uptake ranged from 24% to 36% for diabetic people from 2007 to 2016 [10]. Results from the U.K. bring higher rates, between 63.1% and 69% in persons with diabetes [37].

One of the most relevant findings of our investigation was that influenza vaccine uptake did not improve among diabetic patients from 2011 to 2020. Possible reasons for this lack of improvement may be related to the public discussion about the effectiveness, safety and necessity of influenza vaccination after the 2009 pandemic. Accordingly, a decline in influenza vaccine uptake in recent years has been observed in other European countries [38,39,40,41,42,43,44,45]. Zürcher et al. reported that influenza vaccination uptake among the diabetic population in Switzerland dropped from 41.5% in 2007 to 33.2% in 2017 [42].

When we analysed predictors of influenza vaccination uptake, age was the most important predictor of uptake. The likelihood of diabetic subjects aged 65–74 years being vaccinated was more than fourfold compared to the 18–49 age group, and almost sevenfold if diabetes patients were aged 75 years or over. Other studies have reported similar results [21,22]. This association can be because in Spain, influenza vaccine recommendation is universal for all people aged 65 years or over [46,47]. In addition, multivariable adjustment showed that female gender was associated with lower likelihood of being vaccinated among those reporting diabetes. We agree with a recent study conducted in Spain showing that male gender was a positive predictor of influenza vaccination uptake in diabetic subjects [19]. Possible explanations for this gender difference are that women have less social support, differences in health status and provider bias [48,49].

In our population, the presence of self-reported chronic conditions increased the uptake, as has been reported by other studies [50,51,52]. Suffering respiratory diseases was a positive predictor of vaccine uptake among diabetic adults living in Spain in EHS 2020. The fact that respiratory diseases are closely involved in influenza complications, and vaccination is thus strongly recommended in this high-risk group, may explain this association [53,54,55,56]. Diabetic participants with self-reported cancer also showed higher vaccine uptake. Influenza vaccination is recommended for cancer patients, cancer survivors and those who are not receiving cancer treatment, since influenza vaccination is proven to provide protective immunity against severe infections at similar rates to healthy individuals, reducing morbidity and mortality [57,58,59].

Regarding lifestyle variables, we found that physically active and non-smokers reported higher uptake rates for influenza vaccination, in agreement with other studies [60,61,62].

The low influenza vaccination uptake among the diabetic population found in the present work means it is necessary to implement new strategies with the aim of improving uptake. Jiménez-García et al. interviewed 2288 type 2 diabetes mellitus patients living in Madrid (Spain) regarding their influenza vaccination status and reasons for not receiving the vaccine. Their results showed that the most common reasons for refusing influenza vaccination were the perception of not being at risk for influenza complications and fear of adverse vaccine reactions [49]. Based on this study and other investigations conducted among other high-risk populations, we consider that strategies aiming to improve levels of influenza vaccination among the diabetic population should be mainly focused on healthcare workers and diabetic patients. Healthcare workers should take all available opportunities in their interactions with diabetic patients to provide objective information about the risks of influenza and the benefits of the influenza vaccine. Patient-level interventions that have proved effective among high-risk patients and should be considered include sending postcards, personalized phone calls, emails, home visits and educational interventions for persons with diabetes and their relatives on the risks of influenza and the need for vaccination [46,48,49,63,64,65,66,67,68,69].

The main strengths of this work are the large sample analysed, which is representative at a national level, and the possibility of analysing socio-demographic and lifestyles variables that are not usually available in clinical histories. However, our study also has several limitations. First, data from EHS and SNHS may be affected by non-response, memory and/or social desirability bias. Second, as we do not have information on the reasons for not receiving the vaccine and we cannot make safe and concrete suggestions about the specific strategies to improve uptake in this high-risk population. It would be desirable to include, in future surveys, questions concerning why some diabetic persons do not comply with this preventive measure. Third, to our knowledge, there is no evaluation on the validity of the questions used by the surveys we consulted to classify subjects as diabetic or non-diabetic. However, other authors have reported the high sensitivity and specificity for self-reported diabetes when compared to medical records [70]. Finally, the use of a cross-sectional design means that causality cannot be inferred, as “reverse causality” must be considered.

## 5. Conclusions

Influenza vaccine uptake is below desirable levels among the adult diabetic population in Spain and has not improved between 2011 and 2020. More efforts should be made to increase influenza vaccine uptake in this high-risk group, especially for women, those aged 18–64 years, those without other high-risk conditions, and smokers.

## Figures and Tables

**Table 1:** Vaccine uptake across the surveys according to age and gender.

VARIABLES	SNHS 2011			EHS 2014			SNHS 2017			EHS 2020			TOTAL		
	DM	NO DM	*p*-Value	DM	NO DM	*p*-Value	DM	NO DM	*p*-Value	DM	NO DM	*p*-Value	DM	NO DM	*p*-Value
	*N* (%)	*N* (%)		*N* (%)	*N* (%)		*N* (%)	*N* (%)		*N* (%)	*N* (%)		*N* (%)	*N* (%)	
Age Groups (Years)															
18–49	34 (20.2)	9 (5.4)	<0.001	42 (24.1)	9 (5.2)	<0.001	32 (20.8)	8 (5.2)	<0.001	35 (25.7)	9 (6.6)	<0.001	143 (22.6)	35 (5.5)	<0.001
50–64	188 (36.4)	98 (19)	<0.001	183 (37.9)	90 (18.6)	<0.001	197 (37.6)	86 (16.4)	<0.001	150 (29.5)	69 (13.6)	<0.001	718 (35.3)	343 (16.9)	<0.001
65–74	285 (60.9)	227 (48.5)	<0.001	322 (58.5)	254 (46.2)	<0.001	375 (55.9)	281 (41.9)	<0.001	363 (57.6)	270 (42.9)	<0.001	1345 (58.0)	1032 (44.5)	<0.001
75 or older	425 (68.5)	406 (65.5)	0.251	469 (70.6)	433 (65.2)	0.034	526 (68.4)	491 (64)	0.069	519 (67.1)	484 (62.5)	0.066	1939 (68.6)	1814 (64.2)	<0.001
Gender															
Male	505 (52)	444 (45.7)	0.006	538 (54.9)	447 (45.6)	<0.001	572 (53.3)	461 (42.9)	<0.001	568 (55)	431 (41.8)	<0.001	2183 (53.8)	1783 (43.9)	<0.001
Female	427 (53.2)	296 (36.9)	<0.001	478 (53.6)	339 (38)	<0.001	558 (53.4)	405 (38.9)	<0.001	499 (49.1)	401 (39.4)	<0.001	1962 (52.3)	1441 (38.4)	<0.001
Total Per Survey	932 (52.6)	740 (41.7)	<0.001	1016 (54.3)	786 (42.0)	<0.001	1130 (53.4)	866 (40.9)	<0.001	1067 (52.1)	832 (40.6)	<0.001	4145 (53.1)	3224 (41.3)	<0.001

DM: individuals with diabetes mellitus. NO DM: individuals without diabetes mellitus. SNHS 2011: Spanish National Health Survey 2011. EHS 2014: European Health Survey 2014. SNHS 2017: Spanish National Health Survey 2017. EHS 2020: European Health Survey 2020.

**Table 2:** Vaccine uptake in European Health Survey 2020 (EHS 2020).

Variables	Categories	DM		NO DM		*p*-Value
		*N* (%)	95% CI	*N* (%)	95% CI	
Marital status	Single	91 (38.2)	(32.0–44.7)	64 (26.6)	(21.1–32.6)	0.006
	Married	577 (51.9)	(48.9–54.9)	456 (40.5)	(37.6–43.5)	<0.001
	Divorced, separated, or widowed	**398 (57.0)**	(53.3–60.7)	310 (45.7)	(41.9–49.6)	<0.001
Educational level	Primary	**678 (59.1)**	(56.2–62.0)	491 (51.9)	(48.7–55.1)	0.001
	Secondary	284 (44.6)	(40.7–48.5)	217 (31.0)	(27.5–34.5)	<0.001
	Higher	105 (39.6)	(33.7–45.8)	124 (30.8)	(26.4–35.6)	0.019
Social status	Upper	98 (43.0)	(36.5–49.7)	111 (32.5)	(27.5–37.7)	0.011
	Medium	**347 (53.1)**	(49.2–57.0)	258 (39.3)	(35.6–43.2)	<0.001
	Lower	552 (52.6)	(49.5–55.6)	397 (43.4)	(40.2–46.7)	<0.001
Obesity	No	684 (51.4)	(48.7–54.1)	616 (39.4)	(37.0–41.9)	<0.001
	Yes	**299 (52.4)**	(48.2–56.5)	146 (42.4)	(37.2–47.9)	0.004
Smoking habits	No	**966 (55.4)**	(53.0–57.7)	762 (44.3)	(42.0–46.7)	<0.001
	Yes	101 (33.2)	(28.0–38.8)	67 (20.6)	(16.3–25.4)	<0.001
Alcohol consumption	No	**720 (55.0)**	(52.3–57.8)	518 (44.2)	(41.3–47.1)	<0.001
	Yes	347 (47.0)	(43.4–50.7)	312 (35.7)	(32.5–39.0)	<0.001
Physical activity	No	**532 (53.1)**	(50.0–56.3)	371 (43.2)	(39.9–46.6)	<0.001
	Yes	535 (51.0)	(48.0–54.1)	461 (38.7)	(35.9–41.5)	<0.001
Self-Rated Health	Very good or good	**436 (53.4)**	(49.9–56.8)	425 (42.3)	(39.2–45.4)	<0.001
	Regular	407 (52.5)	(48.6–55.7)	274 (40.1)	(36.4–43.9)	<0.001
	Poor or very poor	224 (49.6)	(44.9–54.3)	133 (36.8)	(31.9–42.0)	<0.001
Myocardial infarction	No	976 (51.4)	(49.1–53.7)	788 (40.1)	(37.9–42.3)	<0.001
	Yes	**91 (60.7)**	(52.4–68.5)	44 (52.4)	(41.2–63.4)	0.218
Respiratory diseases	No	898 (50.3)	(47.9–52.6)	720 (38.8)	(36.6–41.1)	<0.001
	Yes	**169 (64.3)**	(58.1–70.1)	112 (57.7)	(50.4–64.8)	0.156
Cancer	No	948 (50.5)	(48.2–52.8)	755 (40.1)	(37.9–42.3)	<0.001
	Yes	**119 (68.8)**	(61.3–75.6)	77 (46.7)	(38.9–54.6)	<0.001
Stroke	No	984 (51.2)	(48.9–53.5)	798 (40.3)	(38.1–42.5)	<0.001
	Yes	**83 (65.4)**	(56.4–73.6)	34 (50.0)	(37.6–62.4)	0.037

DM: diabetes mellitus. CI: confidence interval. The categories with the highest values of influenza vaccination uptake among the DM population are highlighted with bold font.

**Table 3:** Multivariable analysis for vaccination trends among individuals with diabetes included in SNHS 2011, EHS 2014, SNHS 2017 and EHS 2020.

Variables	Categories (*N*)	OR (95% CI)
Age groups (years)	18–49 (565)	1
	50–64 (1805)	1.757 (1.402–2.202)
	64–74 (2057)	4.197 (3.333–5.285)
	75 or older (2412)	6.753 (5.300–8.604)
Gender	Male (3370)	1
	Female (3469)	0.872 (0.772–0.982)

OR: odds ratio. CI: confidence interval. The value of the Hosmer–Lemeshow goodness-of-fit statistic was 10.04 and the corresponding *p*-value was 0.186. This indicates that the differences between predicted and observed results are not significantly different, so the model fits correctly.

**Table 4:** Multivariable analysis for vaccine uptake in EHS 2020 for diabetic patients.

Variables	Categories (*N*)	OR (95% CI)
Age groups (years)	18–49 (135)	1
	50–64 (507)	1.115 (0.717–1.733)
	65–74 (630)	3.38 (2.177–5.247)
	75 or older (774)	5.102 (3.233–8.051)
Smoking habits	No (1743)	1
	Yes (303)	0.63 (0.474–0.837)
Physical activity	No (1308)	1
	Yes (738)	1.289 (1.052–1.579)
Respiratory diseases	No (1783)	1
	Yes (263)	1.784 (1.330–2.394)
Cancer	No (1873)	1
	Yes (173)	1.672 (1.170–2.388)

OR: odds ratio. CI: confidence interval. The value of the Hosmer–Lemeshow goodness-of-fit statistic was 12.36 and the corresponding *p*-value was 0.089. This indicates that the differences between predicted and observed results are not significantly different, so the model fits correctly.

## Data Availability

According to the contract signed with the Spanish Ministry of Health and Social Services, which provided access to the databases from Spanish National Health Survey and European Health Survey for Spain, we cannot share the databases with any other investigator, and we have to destroy the databases once the investigation has concluded. Consequently, we cannot upload the databases to any public repository. However, any investigator can apply for access to the databases by filling out the questionnaire available at http://www.msssi.gob.es/estadEstudios/estadisticas/estadisticas/estMinisterio/SolicitudSNHSdocs/Formulario_Peticion_Datos_SNHS.pdf. [accessed 14 November 2021]. All other relevant data are included in the paper.

## Supplementary Materials

The following are available online at https://www.mdpi.com/article/10.3390/jcm11010068/s1, Table S1. Definition of study variables used in our investigation according to the questions included in the European Health Survey for Spain 2020 and 2014 and the Spanish National Health Interview Surveys for years 2017 and 2012. Table S2. Distribution of study population according to study variable in patients with and without diabetes in Spain. European Health Survey 2020 (EHS2020).
