# Supplementary material for: Low Levels of Influenza Vaccine Uptake among the Diabetic Population in Spain: A Time Trend Study from 2011 to 2020
Source: J Clin Med. 2021 Dec 23;11(1):68. doi: 10.3390/jcm11010068 (PMC8745480; doi:10.3390/jcm11010068)
Supplement: Supplementary file 1 [file jcm-11-00068-s001.zip › jcm-1500323-supplementary.pdf]

Table S1. Definition of study variables used in our investigation according to the questions included in the European Health Survey for Spain 2020 and 2014 and the Spanish National Health Interview Surveys for years 2017 and 2012.

QUESTIONS	DESCRIPTION AND ANSWER	VARIABLE NAME	CATEGORIES
Were you vaccinated against influenza during the last vaccination campaign?	Those that answered "Yes" were considered vaccinated.	Influenza vaccination	No<br>Yes
Which is your sex?	Man<br>Woman	Gender	Male: when answered "man".<br>Female: when answered "woman".
How old are you?	Age in years	Age groups	18-54<br>55-64<br>65-74<br>75 or older
What is your legal civil status?	1. Single<br>2. Married<br>3. Widower<br>4. Separated<br>5. Divorced	Marital status	Single: option 1<br>Married: option 2<br>Other: option 3,4 and 5.
Please, indicate the highest level of education you have completed.	1. Does not know how to read or write<br>2. Incomplete primary education<br>3. Complete primary education<br>4. First stage of Secondary Education, with or without a qualification<br>5. Elementary Spanish Upper Secondary Education<br>6. Upper secondary education<br>7. Intermediate vocational training or equivalent<br>8. Advanced vocational training or equivalent<br>9. University studies or equivalent<br>10. Over university (master, PhD….)	Educational level	1. Primary school or less: Options 1 to 3<br>2. Secondary school or equivalent: Options 4 to 8<br>3. Higher education: Options 9 and 10
What occupation, profession or job do you perform in your actual position?	Occupations are coded following the National Classification of Occupations 2011 (CNO2011)	Social status	1. Upper<br>2. Medium<br>3. Lower
#1. Could you tell me what your height is, without shoes?	Body mass index is calculated with height and weight	Obesity	1. Yes: If body mass index is 30 or over

#2. Could you tell me how much you weigh, without clothes or shoes?			2. No: If body mass index is under 30
Could you tell me if you smoke?	1. Yes, I smoke daily<br>2. Yes, I smoke, but not daily<br>3. I don't currently smoke but have smoked before<br>4. I neither smoke nor have I ever smoked regularly	Smoking habits	1. Yes: Options 1 and 2<br>2. No: Options 3 and 4
During the past 12 months, how often have you had alcoholic beverages of any kind (i.e. beer, wine, spirits, distilled and mixed drinks, or other alcoholic beverages)?	1. Daily or almost daily<br>2. 5-6 days per week<br>3. 3-4 days per week<br>4. 1-2 days per week<br>5. 2-3 days in a month<br>6. Once a month<br>7. Less than once a month<br>8. Not in the last 12 months, have I stopped drinking<br>9. Never or just a few sips to taste it throughout life	Alcohol consumption	1. Yes: Options 1 to 6<br>2. No: Option 7 to 9
Which of these possibilities best describes how often you do some physical activity in your free time?	1. I don't exercise. I occupy my free time almost completely sedentary<br>2. I do some occasional physical or sports activity<br>3. I do physical activity several times a month<br>4. I do sports or physical training several times a week	Physical activity	1. No: Option 1<br>2. Yes: Option 2 to 4
In the last 12 months, how would you rate your health?	1. Very good<br>2. Good<br>3. Fair<br>4. Bad<br>5. Very bad	Self-rated Health	1. Very good/good: Options 1 and 2<br>2. Fair/poor/very poor: Options 3 to 5<br>1. Very good/good: Options 1 and 2
#1.Do you have, or have you ever had any of the following diseases or medical conditions? Those who answered "yes" completed question #2<br><br>*#2.Have you suffered from that disease/medical condition over the last 12 months? Those who answered "yes" completed question #3*<br><br>*#3. Have you been diagnosed by a physician with this disease/health condition?*	A list of 32 conditions was given to the interviewee including: myocardial infarction	Myocardial infarction	Yes: when answered affirmatively to the question #3 for myocardial infarction.<br>No: any other given answer
	A list of 32 conditions was given to the interviewee including: asthma, emphysema, chronic obstructive pulmonary disease and/or chronic bronchitis.	Respiratory diseases	Yes: when answered affirmatively to the question for any of the listed chronic conditions.<br>No: any other given answer.
	A list of 32 conditions was given to the interviewee including Diabetes Mellitus.	Diabetes Mellitus	Yes: when answered affirmatively to the question #3 for diabetes mellitus.<br>No: any other given answer

A list of 32 conditions was given to the interviewee including: Malignant Tumors	Cancer	Yes: when answered affirmatively to the question for Malignant Tumors.<br><br>No: any other given answer.
A list of 32 conditions was given to the interviewee including: stroke	Stroke	Yes: when answered affirmatively to the question for Stroke.<br><br>No: any other given answer

Table S2. Distribution of study population according to study variable in patients with and without diabetes in Spain. European Health Survey 2020 (EHS2020).

VARIABLES		DM	NO DM	TOTAL	P-value
		N (%)	N (%)	N (%)	
**Age groups (years)**					
	18-49	136 (6.60)	136 (6.60)	272 (6.60)	1
	50-64	509 (24.80)	509 (24.80)	1018 (24.80)	1
	65-74	630 (30.70)	630 (30.70)	1260 (30.70)	1
	75 or older	774 (37.80)	774 (37.80)	1548 (37.80)	1
**Gender**					
	Male	1032 (50.40)	1032 (50.40)	2064 (50.40)	1
	Female	1017 (49.60)	1017 (49.60)	2034 (49.60)	1
**Social status**					
	Upper	228 (11.80)	342 (17.90)	570 (13.90)	<0.001
	Medium	653 (33.80)	656 (34.30)	1309 (31.90)	0.956
	Lower	1050 (54.40)	914 (47.80)	1964 (47.90)	0.002
**Marital status**					
	Single	238 (11.60)	241 (11.80)	479 (11.70)	0.927
	Married	1112 (54.30)	1125 (55.00)	2237 (54.60)	0.800
	Other	698 (34.10)	678 (33.20)	1376 (33.60)	0.609
**Educational level**					
	Primary	946 (46.20)	1147 (56.00)	2093 (51.10)	<0.001
	Secondary	701 (34.20)	637 (31.10)	1338 (32.70)	0.085
	Higher	402 (19.60)	265 (12.90)	667 (16.30)	<0.001
**Obesity**					
	No	1330 (70.00)	1562 (82.00)	2892 (70.60)	<0.001
	Yes	571 (30.00)	344 (18.00)	915 (22.30)	<0.001
**Physical activity**					
	No	1001 (48.90)	858 (41.90)	1859 (45.40)	0.001
	Yes	1048 (51.10)	1191 (58.10)	2239 (54.60)	0.003
**Smoking habits**					
	No	1745 (85.20)	1719 (84.10)	3464 (84.60)	0.671
	Yes	304 (14.80)	325 (15.90)	629 (15.40)	0.425
**Alcohol consumption**					
	No	1308 (63.90)	1172 (57.30)	0 (0.00)	0.007
	Yes	738 (36.10)	874 (42.70)	1612 (39.40)	0.001
**Self-Rated Health**					
	Very good or good	817 (39.90)	1005 (49.00)	1822 (44.50)	<0.001
	Regular	780 (38.10)	683 (33.30)	1463 (35.70)	0.012
	Poor or very poor	452 (22.10)	361 (17.60)	813 (19.80)	0.002
**Myocardial infarction**					
	No	1899 (92.70)	1965 (95.90)	3864 (94.30)	0.296
	Yes	150 (7.30)	84 (4.10)	234 (5.70)	<0.001
**Respiratory diseases**					
	No	1786 (87.20)	1855 (90.50)	3641 (88.80)	0.26
	Yes	263 (12.80)	194 (9.50)	457 (11.20)	0.001
**Cancer**					
	No	1876 (91.60)	1884 (91.90)	3760 (91.80)	0.909
	Yes	173 (8.40)	165 (8.10)	338 (8.20)	0.703
**Stroke**					
	No	1922 (93.80)	1981 (96.70)	3903 (95.20)	0.353
	Yes	127 (6.20)	68 (3.30)	195 (4.80)	<0.001

DM: Individuals with Diabetes Mellitus. NO DM: Individuals without Diabetes Mellitus.
